# Divergent Cardiac Adaptations in Endurance Sport: Atrial Fibrillation Markers in Marathon Versus Ultramarathon Athletes

**DOI:** 10.3390/jcdd12070260

**Published:** 2025-07-07

**Authors:** Zbigniew Waśkiewicz, Eduard Bezuglov, Oleg Talibov, Robert Gajda, Zhassyn Mukhambetov, Daulet Azerbaev, Sergei Bondarev

**Affiliations:** 1 Institute of Sport Sciences, Jerzy Kukuczka Academy of Physical Education, 40-065 Katowice, Poland; 2 Department of Sports Medicine and Medical Rehabilitation, Sechenov First Moscow State Medical University, 119991 Moscow, Russia; e.n.bezuglov@gmail.com; 3 Department of Internal Medicine, Clinical Pharmacology and Emergency Medicine, Moscow State University of Medicine and Dentistry, 127473 Moscow, Russia; oleg.talibov@gmail.com; 4 Center for Sports Cardiology, Gajda-Med Medical Center, 06-100 Pułtusk, Poland; r.gajda@gajdamed.pl; 5 Department of Kinesiology and Health Prevention, Jan Dlugosz University in Częstochowa, 42-200 Częstochowa, Poland; 6 Department of Sport Education and Coaching, Academy of Physical Education and Mass Sport, 010000 Astana, Kazakhstan; zh_mukhambet@apems.edu.kz (Z.M.); d_azerbayev@apems.edu.kz (D.A.); 7 Department of Cardiac, Thoracic, and Vascular Sciences, University of Padova, 35122 Padova, Italy; sergei.bondarev@unipd.it

**Keywords:** atrial fibrillation, endurance athletes, marathon, ultramarathon, cardiac remodeling

## Abstract

Endurance training induces significant cardiac remodeling, with evidence suggesting that prolonged high-intensity exercise may increase the risk of atrial fibrillation (AF). However, physiological responses differ by event type. This review compares AF-related markers in marathon and ultramarathon runners, focusing on structural adaptations, inflammatory and endothelial biomarkers, and the incidence of arrhythmias. A systematic analysis of 29 studies revealed consistent left atrial (LA) enlargement in marathon runners linked to elevated AF risk and fibrosis markers such as Galectin-3 and PIIINP. In contrast, ultramarathon runners exhibited right atrial (RA) dilation and increased systemic inflammation, as indicated by elevated high-sensitivity C-reactive protein (hs-CRP) and soluble E-selectin levels. AF incidence in marathoners ranged from 0.43 per 100 person-years to 4.4%, while direct AF incidence data remain unavailable for ultramarathon populations, highlighting a critical evidence gap. These findings suggest distinct remodeling patterns and pathophysiological profiles between endurance disciplines, with implications for athlete screening and cardiovascular risk stratification.

## 1. Introduction

Endurance exercise provides substantial cardioprotective effects; however, chronic exposure to high-volume training can lead to structural and electrical changes in the myocardium, predisposing athletes to atrial fibrillation (AF) [1,2]. Emerging data indicate modality-specific remodeling of the atria and ventricles among competitive long-distance athletes, particularly those engaged in marathon and ultramarathon events. Marathon runners frequently demonstrate progressive left atrial (LA) enlargement and altered myocardial strain. While partly physiological, these adaptations may act as substrates for atrial arrhythmias, particularly in athletes with high cumulative training volumes [3,4]. Elevated circulating biomarkers of fibrosis and endothelial activation—such as Galectin-3, PIIINP, and sVCAM-1—have also been identified in this population [5,6]. In contrast, ultramarathon runners often exhibit pronounced right atrial (RA) remodeling, characterized by reduced RA strain and increased chamber volume [7]. These changes reflect prolonged hemodynamic loading and sustained volume overload yet their relationship with AF remains underexplored due to limited incidence data.

This review synthesizes the available literature on structural, functional, and molecular markers of AF in marathon and ultramarathon runners. The aim is to identify key differences in cardiac remodeling and arrhythmogenic risk, thereby guiding screening and training practices in endurance athletes. This review aims not only to summarize current evidence but also to explicitly contrast cardiac remodeling, inflammatory signaling, and arrhythmia risk between marathon and ultramarathon disciplines, identifying divergence in AF-related markers.

## 2. Methods

### 2.1. Search Strategy

A targeted literature search was conducted using the Semantic Scholar database (https://www.semanticscholar.org/), which indexes over 126 million scholarly articles. The search was performed on 9 January 2025 using the query:

“atrial fibrillation” AND (“marathon runners” OR “ultramarathon runners” OR “endurance athletes”) AND (“left atrial remodeling” OR “right atrial strain” OR “cardiac fibrosis” OR “arrhythmia incidence” OR “biomarkers”)

The aim was to identify peer-reviewed studies reporting structural, functional, or biochemical markers associated with atrial fibrillation (AF) in long-distance endurance runners.

### 2.2. Eligibility Criteria

Studies were included if they met all of the following criteria:

- Involved adult participants (≥18 years old).
- Included populations of marathon (≥42.2 km) or ultramarathon (>42.2 km) runners
- Reported at least one marker associated with AF, including but not limited to atrial size, myocardial strain, P-wave duration, inflammatory or fibrotic biomarkers, or arrhythmia outcomes
- Employed a comparative or observational study design (cross-sectional, case–control, prospective, or retrospective cohort);
- Focused on chronic cardiac adaptations, excluding acute responses immediately after isolated exercise bouts
- Included either a non-running control group or compared different endurance distances
- Published in English-language peer-reviewed journals.

Studies were excluded if they (1) focused solely on recreational joggers or sub-marathon runners, (2) lacked extractable data on cardiac or electrophysiological markers, or (3) involved participants with known cardiovascular disease at baseline.

### 2.3. Study Screening and Selection

Title and abstract screening were conducted independently by one reviewer. A full-text review was performed for studies that met the initial criteria or when eligibility was unclear. Screening decisions were based on a comprehensive review of the study’s aims, participant characteristics, and reported outcomes. A total of 29 studies met all inclusion criteria and were retained for data extraction.

### 2.4. Data Extraction

For each included study, the following data were extracted:

- Study design (e.g., cross-sectional, prospective cohort).
- Sample characteristics (total number, gender distribution, age, training background, event type).
- AF markers: Structural (e.g., LA/RA size, strain), functional (e.g., ECG features), and biochemical (e.g., sVCAM-1, hs-CRP, Galectin-3).
- AF incidence or prevalence, when reported, including hazard ratios (HRs), confidence intervals (CIs), and statistical significance values.
- Measurement techniques (e.g., echocardiography, cardiac MRI, biomarker assay methods).
- Follow-up duration for longitudinal studies.

When multiple outcomes were reported, preference was given to the most comprehensive or statistically adjusted data. Numerical data were extracted directly from tables or text, and units of measurement were standardized when necessary.

### 2.5. Data Synthesis

**Figure 1:**
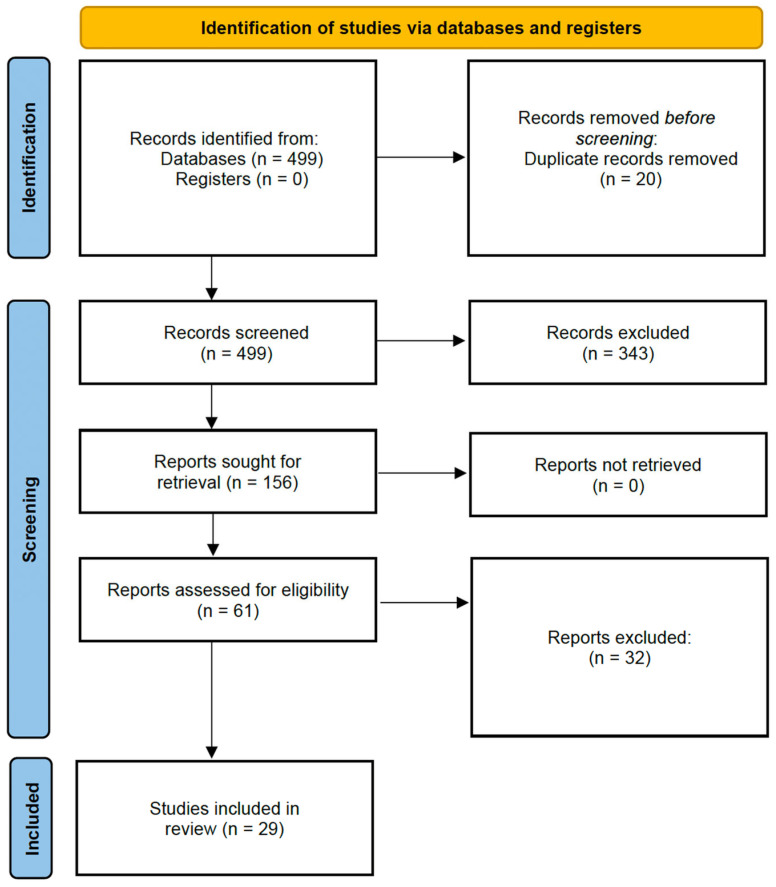
PRISMA flow diagram description.

Due to heterogeneity in study designs, populations, and outcomes, a narrative synthesis approach was applied. Quantitative pooling (meta-analysis) was not performed. Tables were constructed to summarize findings by structural, electrophysiological, and molecular categories. The study selection process followed a structured approach inspired by the PRISMA (Preferred Reporting Items for Systematic Reviews and Meta-Analyses) guidelines [8]. The steps are detailed in Figure 1. The final qualitative synthesis included 29 studies. These studies provided data on structural, electrophysiological, or biomolecular markers of atrial remodeling and AF risk in marathon and/or ultramarathon athletes.

### 2.6. Risk of Bias and Quality Assessment

To assess the methodological quality of the included studies, we applied the Newcastle–Ottawa Scale (NOS), a validated tool for evaluating the quality of non-randomized observational studies in systematic reviews [9]. Each study was independently rated across three domains:

- Selection (maximum 4 points): including the representativeness of the sample and ascertainment of exposure.
- Comparability (maximum 2 points): based on the control of potential confounders such as age, sex, or training level.
- Outcome/Exposure (maximum 3 points): assessing the method and accuracy of outcome ascertainment, duration of follow-up, and reporting transparency.

The maximum achievable score is 9. Among the 29 included studies, the total NOS scores ranged from 6 to 9, with a median score of 7. Most studies (n = 20) scored ≥7, indicating moderate to high methodological quality. A detailed breakdown of the NOS scoring per study is provided in Supplementary Table S1.

To estimate the pooled prevalence of atrial fibrillation (AF) among endurance athletes, we performed a single-arm meta-analysis of proportions using data from three eligible studies. The inclusion criteria required that each study report the number of AF cases and the total sample size within a clearly defined cohort of adult long-distance runners. The selected studies provided consistent diagnostic definitions of AF and studied comparable athletic populations. We calculated the prevalence for each study as the proportion of AF cases in the total sample. Standard errors (SE) for each proportion were computed using the binomial approximation formula:

SE = √[p(1 − p)/n],

where p is the observed prevalence and n is the sample size. A fixed-effect model was used, justified by low heterogeneity in study design and participant characteristics. Pooled prevalence and 95% confidence intervals (CI) were calculated using inverse-variance weighting. Forest plot visualization was constructed to illustrate individual estimates and the overall summary effect.

## 3. Results

### 3.1. Risk of Bias and Methodological Quality

All 29 studies included in the final synthesis were assessed for methodological quality using the Newcastle–Ottawa Scale (NOS). The total NOS scores ranged from 3 to 9, with a mean score of 6.34, a standard deviation of 1.93, and a median of 6.0. The majority of studies (n = 15) received a score of 6, indicating moderate quality, while nine studies achieved the maximum score of 9, reflecting high methodological rigor. Only four studies scored below 5, primarily due to limited comparability between groups or insufficient reporting of outcomes. A complete breakdown of the quality scores by domain (Selection, Comparability, Outcome/Exposure) is provided in Supplementary Table S1.

### 3.2. Cardiac Structural Adaptations

#### 3.2.1. Atrial Remodeling

Left and right atrial remodeling were consistently observed across endurance athlete subgroups, though patterns varied by training modality (Table 1). Marathon runners demonstrated an increase in left atrial (LA) volume relative to controls, often correlating with cumulative training exposure. Wilhelm et al. [3] reported significantly enlarged LA volumes in marathon runners (42 ± 8 mL/m^2^) compared to sedentary individuals (25 ± 9 mL/m^2^, *p* = 0.001). Similarly, Gabrielli et al. [5] observed higher absolute LA volumes in marathoners (56 ± 13 mL vs. 49 ± 10 mL, *p* = 0.001). Progressive LA enlargement, accompanied by repeated marathon participation, was also documented [10].

In contrast, ultramarathon runners exhibited prominent right atrial (RA) remodeling. Ujka et al. [7] reported more significant RA volumes and reduced RA global longitudinal strain (GLS) in ultramarathoners compared to marathoners (*p* < 0.05), reflecting prolonged right-sided hemodynamic load.

Mixed-endurance cohorts also confirmed LA structural changes as predictive markers for AF. Clauss et al. [11] and Cipriani et al. [12] demonstrated that increased LA diameter and volume were significantly associated with elevated AF risk, even after controlling for age and training duration.

#### 3.2.2. Ventricular Remodeling

Endurance training also produces ventricular adaptations, with modality-specific differences between marathon and ultramarathon athletes (Table 2). Multiple studies have observed an increased left ventricular (LV) mass index among marathon runners. Li et al. [13] reported significantly greater LV mass index in marathon runners (121.1 ± 15.5 g/m^2^) compared to controls (81.8 ± 11.1 g/m^2^, *p* < 0.01). Additionally, reduced myocardial strain of the interventricular septum was noted in this cohort, suggesting early signs of mechanical dysfunction or compensatory remodeling.

Ultramarathon athletes demonstrated distinct right ventricular (RV) adaptations. Picco et al. [14] found increased RV end-diastolic area (*p* = 0.026), while Shin et al. [15] reported elevated RV fractional area change (FAC) post-race (*p* = 0.008). These findings reflect volume-dependent RV remodeling, potentially due to the prolonged physiological demands of ultra-endurance activity.

Breuckmann et al. [16] identified a significant association between increased LV mass and myocardial fibrosis among mixed endurance groups. Athletes with late gadolinium enhancement (LGE) on cardiac MRI had greater LV mass (86 ± 18 g/m^2^) than those without fibrosis (73 ± 14 g/m^2^, *p* < 0.05), raising concerns about cumulative structural injury in long-term endurance athletes.

**Table 2:** Ventricular remodeling in endurance athletes.

Category	Sample Size	LV Change	RV Change	Clinical Relevance
Marathon runners	~100–160 [1,5]	↑ LVEDV and LV mass (*p* < 0.05) [5]	No acute dilation or dysfunction [1]	Adaptive remodeling without dysfunction
Ultramarathon runners	~60–80 [7,17]	Transient ↓ LV EF post-race (*p* < 0.01) [17]	↑ RVEDV; ↓ RV FAC and strain (*p* < 0.01) [7]	RV strain and temporary systolic impairment
Mixed endurance cohorts	100–200 [11,12]	↑ LV mass indexed to BSA [12]	Not consistently reported	Remodeling may affect atrial stretch/load

Note: ↑ = increase; ↓ = decrease.

### 3.3. Functional Markers and Arrhythmia Incidence

Atrial fibrillation (AF) and related arrhythmic markers were predominantly studied in marathon runners (Table 3). The reported incidence of AF ranged from 0.43 cases per 100 person-years [1] to 4.4% in female endurance athletes [18,19]. Wilhelm et al. [2] found a 3.3% incidence of AF exclusively among male non-elite marathon runners, with statistical significance (*p* = 0.042). Furthermore, Haeusler et al. [20] estimated an AF prevalence of 5.1–9.8% in experienced marathoners, suggesting higher rates than in the general population.

Cipriani et al. [12] reported that 17% of marathon athletes demonstrated frequent premature atrial beats (PABs), though this was not significantly different from sedentary controls (22%, *p* = 0.61). While electrical instability is observed in endurance athletes, not all subclinical findings translate into increased AF risk.

Data specific to ultramarathon runners remains sparse. No included study provided incidence or prevalence estimates of AF in this population. However, the remodeling of right-sided structures and elevated inflammatory markers suggest a potential for arrhythmic development, albeit through mechanisms different from those of marathon runners.

**Figure 2:**
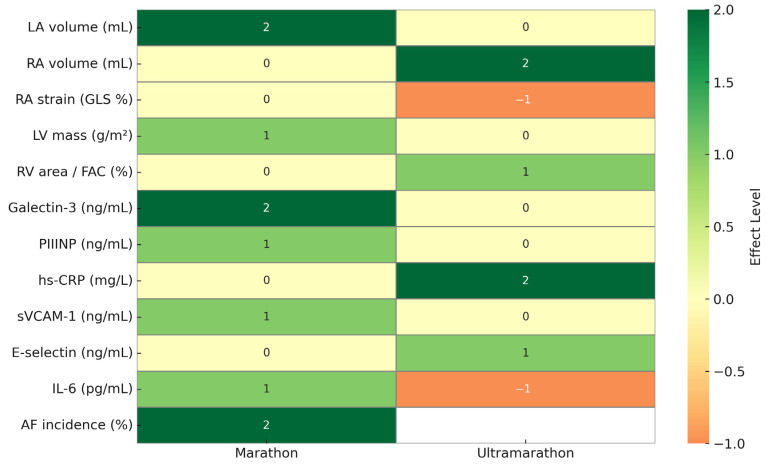
Heatmap summary of atrial fibrillation-related markers in marathon and ultramarathon runners. Effect levels represent the direction and consistency of changes across multiple studies: −1 = reduction, 0 = no change or not reported, 1 = modest increase (1 study), and 2 = consistent increase (≥2 studies). LA, left atrium; RA, right atrium; LV, left ventricle; RV, right ventricle; GLS, global longitudinal strain; FAC, fractional area change.

A heatmap summary (Figure 2) synthesizes the key differences in structural, functional, and biochemical markers of atrial fibrillation (AF) between marathon and ultramarathon runners. Marathon runners exhibit consistent increases in left atrial volume, Galectin-3, and AF incidence, while ultramarathon runners show more pronounced right atrial remodeling, elevated hs-CRP, and increased E-selectin levels. Notably, RA strain is reduced in ultramarathoners, whereas microvascular and inflammatory markers display distinct trends by event type. This visual overview highlights the modality-specific nature of AF risk profiles in endurance athletes.

### 3.4. Biomarker Profiles

#### 3.4.1. Endothelial Function

Endurance exercise affects endothelial homeostasis, with specific biomarkers reflecting vascular stress and atrial remodeling (Table 4). In marathon runners, soluble vascular cell adhesion molecule-1 (sVCAM-1) levels significantly increased post-race—from 651 ± 350 to 905 ± 373 ng/mL (*p* = 0.002)—and were positively correlated with left atrial volume (rho = 0.510, *p* = 0.001) [6]. A separate study reported similar associations with VCAM-1, which was strongly correlated with LA size (rho = 0.483, *p* = 0.007) [5].

In contrast, data from ultramarathon runners highlighted an increase in soluble E-selectin, especially in individuals with exercise-induced hypertension (EIH), indicating systemic endothelial activation [21]. These findings suggest that marathon runners primarily exhibit LA-associated endothelial stress, while ultramarathoners may show more generalized vascular inflammation.

#### 3.4.2. Inflammatory Markers

Marathon runners exhibit increased post-race levels of fibrosis-associated inflammatory markers (Table 5). Galectin-3, a marker of cardiac fibrosis, rose significantly from 6.8 ± 2.2 to 19.7 ± 4.9 ng/mL (*p* = 0.012) post-marathon [22]. Similarly, levels of the amino-terminal propeptide of type III procollagen (PIIINP) increased from 61 ± 16 to 94 ± 24 ng/mL (*p* = 0.01), particularly in less-trained runners from the same cohort.

Ultramarathon runners, while lacking data on Galectin-3 and PIIINP, demonstrated elevated high-sensitivity C-reactive protein (hs-CRP) levels throughout the race and at three days post-race [15], indicating sustained systemic inflammation. Interestingly, IL-6 levels were lower in ultrarunners than in controls (17% vs. 56% with elevated IL-6, *p* < 0.05), which may reflect long-term adaptation to repeated inflammatory stress [21].

Galectin-3 has been implicated in myocardial fibrosis through the activation of fibroblasts and the promotion of TGF-β signaling, contributing to atrial stiffening and electrical heterogeneity [23]. PIIINP reflects collagen turnover and may indicate active extracellular matrix remodeling. Circulating microRNAs have been shown to modulate gene expression linked to atrial hypertrophy and may serve as early markers of remodeling propensity.

### 3.5. Long-Term Cardiovascular Implications

Prolonged endurance training induces chronic structural and biochemical changes in the heart that may persist beyond individual races (Table 6). Among marathon runners, atrial remodeling progressed with the number of marathons completed. Wilhelm et al. [3] observed dose-dependent increases in left and right atrial volumes across three groups of runners based on the number of completed races. This remodeling was paralleled by elevations in circulating pro-atrial natriuretic peptide (pro-ANP), a marker of atrial wall stress [10].

Ragab et al. [24] reported a higher prevalence of late gadolinium enhancement (LGE) in male marathon runners compared to female athletes, suggesting a more significant burden of myocardial fibrosis in males. While the clinical implications of LGE remain debated, fibrosis is a known substrate for atrial arrhythmias and may contribute to an increased incidence of AF.

Data are limited for ultramarathon runners. However, Ujka et al. [25] demonstrated that right atrial remodeling was more pronounced in ultramarathoners than in marathoners. This observation reinforces the notion that distinct atrial loading conditions shape structural outcomes in various endurance modalities. Drca et al. [19] identified a significant increase in long-term AF risk in female endurance athletes (HR = 3.63; 95% CI: 1.56–8.41), confirming that structural and electrophysiological adaptations may translate into clinically significant arrhythmias over time.

### 3.6. Risk Stratification

Identifying athletes at heightened risk of atrial fibrillation (AF) is critical for early intervention and long-term health management in endurance populations (Table 7). Several structural, electrical, and biochemical markers have been proposed as tools for risk stratification. Among marathon runners, left atrial (LA) size emerged as a consistent predictor of AF. Molina et al. [1] demonstrated that increased LA diameter was independently associated with AF incidence in male endurance athletes. Wilhelm et al. [2] have highlighted that the training volume itself is a significant risk factor. Drca et al. [18] reported a positive correlation between cumulative endurance exposure and AF risk in female athletes, reinforcing the need to consider training history when evaluating cardiovascular risk. Emerging evidence also suggests a role for molecular biomarkers in AF prediction. Clauss et al. [11] identified correlations between transcriptomic profiling patterns and LA diameter in elite marathon runners, suggesting a possible future role for transcriptomic profiling in athlete screening.

In contrast, data from ultramarathon runners remains limited. One notable finding from Konwerski et al. [26] was that lower epicardial adipose tissue volume in ultramarathoners was associated with a more favorable cardiovascular profile. While not directly linked to AF incidence, this may reflect protective adaptations in response to extreme endurance exposure. Emerging data point to relevant sex-based differences in remodeling responses. For instance, Ragab et al. [24] observed a higher prevalence of myocardial fibrosis (LGE-positive MRI) among male marathoners, whereas Drca et al. [19] documented an increased incidence of AF in elite female endurance athletes (HR = 3.67; 95% CI: 1.71–7.87) compared to matched population controls. These findings suggest that sex-specific remodeling trajectories may influence arrhythmic vulnerability and should be incorporated into future screening algorithms.

### 3.7. Exploratory Meta-Analysis of Atrial Fibrillation Prevalence

**Figure 3:**
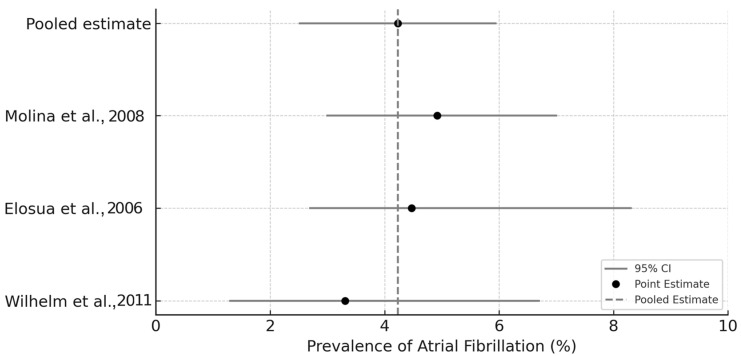
Forest plot of atrial fibrillation prevalence among endurance athletes based on three cohort studies [1,2,28].

The meta-analysis of three independent cohorts yielded a pooled AF prevalence of 4.23% (95% CI: 2.52–5.94%) among endurance athletes (Table 8). The individual study estimates ranged from 3.31% [2] to 4.92% [1], with overlapping confidence intervals and consistent directionality. This finding confirms a reproducible trend of elevated AF risk in this population segment, although absolute prevalence remains below 5% (Table 8, Figure 3).

## 4. Discussion

This review synthesizes evidence from 29 studies comparing atrial fibrillation (AF) markers in marathon and ultramarathon runners. The findings demonstrate distinct remodeling patterns, biochemical signatures, and risk factors across these endurance disciplines. Marathon runners consistently exhibited left atrial (LA) enlargement, which was frequently associated with an increased incidence of atrial arrhythmias and molecular markers of fibrosis. In contrast, ultramarathon runners displayed right atrial (RA) remodeling and elevated systemic inflammatory responses, though direct data on AF incidence in this group remain limited.

### 4.1. Structural Remodeling and Modality-Specific Adaptation

Marathon training induces progressive enlargement of the left atrium, with evidence showing that LA volume increases with training volume and race frequency [3,10]. This anatomical adaptation is a substrate for arrhythmogenesis by facilitating atrial conduction delay and fibrosis. In addition, marathon runners demonstrate increased left ventricular (LV) mass and decreased myocardial strain, further supporting the presence of load-induced myocardial remodeling [13]. Ultramarathon runners, by contrast, more commonly exhibit right atrial enlargement and reduced RA global longitudinal strain [7]. These findings suggest that ultra-endurance exerts a different hemodynamic load profile dominated by sustained venous return and pulmonary circulation stress. Interestingly, ventricular remodeling in ultramarathoners also appears to favor the right side, with increases in right ventricular (RV) end-diastolic area and fractional area change observed in multiple studies [14,15].

### 4.2. Electrophysiological Risk and Arrhythmia Incidence

Among marathon runners, AF incidence ranged from 0.43 per 100 person-years to as high as 4.4%, with hazard ratios indicating a markedly increased risk compared to non-athletic controls [1,18]. Male athletes appear to be particularly susceptible, while female endurance athletes also exhibit an elevated long-term risk [19]. Prolonged P-wave duration and altered sympatho-vagal balance further support the role of electrophysiological remodeling [2]. To synthesize the available evidence, we conducted an exploratory meta-analysis of three original studies that reported the prevalence of atrial fibrillation among endurance-trained athletes. This value aligns with individual study estimates (ranging from 3.3% to 4.9%) and substantially exceeds the prevalence reported in age-matched general population samples, which is typically below 2%. These findings support the hypothesis that chronic endurance exercise may create a sustained substrate for arrhythmogenesis, even in non-elite athletes. Despite pronounced RA remodeling in ultramarathon runners, direct data on AF incidence are lacking. This highlights a critical gap in the literature and suggests the need for long-term, prospective studies in ultra-endurance populations.

### 4.3. Molecular and Inflammatory Pathways

The pathogenesis of AF in endurance athletes is likely multifactorial, involving a complex interplay between hemodynamic load, autonomic modulation, and gene expression. Repeated exposure to high cardiac output during prolonged exercise leads to atrial stretch, increased wall tension, and subsequent extracellular matrix remodeling. Hemodynamic overload may activate profibrotic pathways, including TGF-β signaling and Galectin-3–mediated fibroblast proliferation, ultimately leading to atrial fibrosis and electrical heterogeneity. In parallel, endurance training alters autonomic tone, with parasympathetic predominance at rest (e.g., bradycardia, vagal dominance) and heightened sympathetic drive during effort, potentially contributing to the dispersion of refractoriness. Furthermore, recent data suggest a role for epigenetic regulation in structural adaptation. MicroRNA expression profiles, such as miR-21 and miR-328, have been associated with atrial hypertrophy and conduction delay, and may serve as early biomarkers of maladaptive remodeling.

Biomarker profiles in marathon runners revealed elevations in Galectin-3, PIIINP, and sVCAM-1—markers associated with myocardial fibrosis and endothelial stress [6,22]. These may represent early molecular indicators of maladaptive remodeling. In ultramarathon runners, increased high-sensitivity C-reactive protein (hs-CRP) and soluble E-selectin levels indicate systemic inflammation and endothelial activation [15,21]. Conversely, reduced IL-6 levels in ultramarathoners suggest possible anti-inflammatory adaptations with chronic exposure.

### 4.4. Risk Stratification and Clinical Implications

Risk stratification strategies for endurance athletes should incorporate structural markers such as LA size, P-wave duration, and training history. Emerging biomarkers, such as circulating microRNAs, may contribute to future strategies for individualized AF risk stratification in endurance athletes [11]. In ultramarathon runners, indicators such as epicardial adipose tissue and systemic inflammation may inform risk profiles, although further validation is required. Clinicians, coaches, and sports scientists should be aware of the varying cardiovascular responses across endurance modalities and apply individualized monitoring, particularly in athletes with prolonged training histories or atypical symptoms.

### 4.5. Supporting Observations from Supplementary Studies

Several additional studies included in the quality assessment contributed supporting insights into cardiac remodeling or electrophysiological changes, although they were not part of the primary synthesis. Doni et al. [28] reported an increase in right atrial size among amateur marathon runners, supporting chamber-specific remodeling (NOS = 6). Louis-Georges et al. [29] described ST-segment and T-wave alterations in mountain race athletes. At the same time, Madalosso and Raviele [30,31] documented an elevated incidence of lone AF in male endurance athletes across two related publications. However, stratification by exposure volume was lacking. Maqueda et al. [32] investigated gene expression adaptation in ultra-trail runners, identifying shifts in neurocardiac regulation. Classic Doppler studies, such as those by Martínez-Mas et al. [33], Möckel et al. [34], and Störk et al. [35], provided early evidence of altered left ventricular filling dynamics in endurance athletes, albeit using historical protocols and small sample sizes.

### 4.6. Limitations and Future Study

This review is limited by heterogeneity across study designs, methodologies, and reporting standards. Few studies directly compared marathon and ultramarathon runners within the same dataset. Additionally, sex-specific analyses were limited, and AF incidence was rarely reported in female or ultramarathon cohorts. Finally, the cross-sectional nature of most studies limits the ability to make causal inferences. Significantly, the lack of direct, prospectively collected AF incidence data in ultramarathon runners severely limits risk stratification in this subgroup. While surrogate markers of remodeling and inflammation are frequently reported, their predictive validity for clinical arrhythmia remains unverified.

The analysis of ultramarathon athletes is limited by the scarcity of studies reporting AF as a clinical endpoint. While several investigations have documented RA volumetric expansion, reduced RA strain, and systemic inflammatory activation in response to ultra-distance efforts, these data remain indirect. Without electrocardiographic surveillance or rhythm monitoring, it is not possible to determine whether these structural changes translate into arrhythmogenic risk. This gap compromises comparability with marathon data and highlights the need for longitudinal studies that incorporate arrhythmia tracking in ultra-endurance populations.

Future research should prioritize prospective, longitudinal studies with harmonized definitions of training load, cardiac assessment, and arrhythmia detection. Integrating imaging, electrophysiology, and molecular profiling will be key to refining cardiovascular risk prediction in endurance athletes.

## 5. Conclusions

Marathon and ultramarathon runners exhibit distinct patterns of atrial remodeling and biomarker activation, reflecting differences in physiological load and systemic stress. Marathoners are more prone to left atrial enlargement and fibrosis-related signaling, while ultramarathoners show right-sided remodeling and systemic inflammation. Although AF incidence is elevated in marathoners, this remains poorly quantified in ultramarathon cohorts, underscoring the need for targeted longitudinal research. Risk stratification in endurance athletes should be modality-specific, incorporating structural, electrophysiological, and molecular indicators. These findings highlight the importance of individualized cardiovascular monitoring and training management for athletes participating in prolonged endurance activities. Further research should prioritize mechanistic investigations that integrate hemodynamic loading, autonomic modulation, and molecular signatures—such as microRNAs and fibrosis biomarkers—to enhance early detection and prevention strategies in high-volume endurance athletes.

## Figures and Tables

**Table 1:** Atrial remodeling in endurance athletes.

Category	Sample Size	LA Volume Change	RA Volume Change	Clinical Relevance
Marathon runners	~121–200 [3,5]	↑ 42 ± 8 vs. 25 ± 9 mL/m^2^ (*p =* 0.001) [3]; ↑ 56 ± 13 vs. 49 ± 10 mL (*p =* 0.001) [5]	Not reported/↑ with cumulative exposure [3]	LA enlargement associated with AF risk [10]
Ultramarathon runners	n = 45 [7]	Not reported	↑ RA volume; ↓ RA GLS (*p* < 0.05) [7]	RA remodeling may promote arrhythmogenesis [7]
Mixed endurance cohorts	~134–200 [11,12]	↑ LA diameter/volume associated with AF *(p <* 0.05) [11,12]	Not reported	LA metrics predictive of AF incidence [12]

Note: ↑ = increase; ↓ = decrease.

**Table 3:** Atrial fibrillation and arrhythmic markers in marathon runners.

Study	Sample Size	AF Estimate	Key Arrhythmic Finding	Interpretation
Drca et al. [18,19]	12,045 athletes	4.4% incidence; HR 3.63–3.67	AF risk in elite female endurance athletes	Significantly higher risk compared to matched general population
Molina et al. [1]	1006 marathon runners	0.43/100 PY; HR 8.80 (95% CI: 1.26–61.29)	Long-term training associated with increased AF	Cumulative exposure raises lone AF incidence
Wilhelm et al. [2]	121 non-elite male runners	3.3% incidence; *p* = 0.042	AF in males; none observed in females	Sex-specific vulnerability and atrial remodeling
Cipriani et al. [12]	130 athletes/130 controls	17% PABs vs. 22% in controls; *p* = 0.61	No significant difference in ectopy prevalence	AF surrogate markers not elevated in athletes
Haeusler et al. [20]	Survey-based (estimated *n* > 500)	Estimated prevalence: 5.1–9.8%	Elevated self-reported AF prevalence	Suggests underdiagnosed burden in marathon runners

**Table 4:** Endothelial biomarkers in endurance athletes.

Biomarker	Marathon Runners	Ultramarathon Runners	Interpretation
sVCAM-1	↑ post-marathon (651 ± 350 → 905 ± 373 ng/mL, *p* = 0.002) [6]	Not reported	Associated with LA volume and atrial remodeling
VCAM-1	Correlated with LA volume (rho = 0.483, *p* = 0.007) [5]	Not reported	Potential biomarker for AF risk in trained athletes
Soluble E-selectin	Not reported	↑ in EIH runners [21]	Marker of endothelial activation during ultra-endurance exposure

**Note:** ↑ = increase.

**Table 5:** Inflammatory markers in marathon and ultramarathon runners.

Biomarker	Marathon Runners	Ultramarathon Runners	Interpretation
Galectin-3	↑ post-marathon (6.8 ± 2.2 → 19.7 ± 4.9 ng/mL, *p* = 0.012) [22]	Not reported	Marker of myocardial fibrosis linked to LA remodeling
PIIINP	↑ in less-trained runners (61 ± 16 → 94 ± 24 ng/mL, *p* = 0.01) [22]	Not reported	Reflects collagen turnover and fibrotic signaling
IL-6	Not reported	↓ elevated IL-6 levels vs. controls (17% vs. 56%, *p* < 0.05) [21]	Possible anti-inflammatory adaptation in ultrarunners
hs-CRP	Not reported	↑ throughout race and at day 3 post-race [15]	Indicator of systemic inflammation and physiological stress

Note: ↑ = increase; ↓ = decrease.

**Table 6:** Long-term cardiovascular adaptations in endurance athletes.

Study	Marathon Runners	Ultramarathon Runners	Clinical Implications
Wilhelm et al. [3]	Progressive LA and RA enlargement based on race volume	Not reported	Suggests cumulative remodeling from repetitive endurance exposure
Wilhelm et al. [10]	↑ pro-ANP levels correlated with atrial size	Not reported	Indicates chronic atrial wall stress with endurance training
Ragab et al. [24]	Higher LGE prevalence in male marathoners	Not reported	Suggests subclinical myocardial fibrosis and potential long-term arrhythmic substrate
Drca et al. [19]	HR for AF = 3.63 (95% CI: 1.56–8.41) in female runners	Not reported	Long-term AF risk elevated in female endurance athletes
Ujka et al. [7]	Not reported	Greater RA remodeling than in marathon runners	Suggests endurance-specific remodeling of the right atrium under ultra-endurance conditions

Note: ↑ = increase.

**Table 7:** Risk markers for atrial fibrillation in endurance athletes.

Study	Marathon Runners	Ultramarathon Runners	Interpretation
Molina et al. [1]	LA size positively associated with AF incidence	Not reported	Structural markers such as LA volume are predictive of arrhythmia
Wilhelm et al. [11]	Prolonged P-wave duration; increased sympatho-vagal tone; ↑ LA volume	Not reported	Multiple interacting factors influence AF risk
Drca et al. [18]	Training volume correlates with increased AF risk	Not reported	Highlights cumulative burden of endurance exercise
Clauss et al. [11]	MicroRNA expression associated with LA diameter in elite runners	Not reported	Potential role for epigenetic markers in individualized AF screening
Konwerski et al. [26]	Not reported	Lower epicardial adipose tissue linked to favorable CV profile	May suggest cardioprotective adaptation in ultra-endurance runners

**Note:** ↑ = increase.

**Table 8:** AF prevalence meta-analysis summary.

Study	Design	Sample Size	AF Cases	Prevalence (%)	95% CI
Wilhelm et al. [2]	Cross-Sectional	121	4	3.31	1.3–6.7
Elosua et al. [27]	Case–Control	200	9	4.47	2.7–8.3
Molina et al. [1]	Prospective Cohort	1006	49	4.92	3.0–7.0
Pooled Estimate	Random Effects Model	–	–	4.23	2.52–5.94

## Data Availability

No new data are presented in this narrative review.

## Supplementary Materials

The following supporting information can be downloaded at https://www.mdpi.com/article/10.3390/jcdd12070260/s1, Table S1: Quality assessment of included studies using the Newcastle–Ottawa Scale (NOS).
