# Supplementary material for: Divergent Cardiac Adaptations in Endurance Sport: Atrial Fibrillation Markers in Marathon Versus Ultramarathon Athletes
Source: J Cardiovasc Dev Dis. 2025 Jul 7;12(7):260. doi: 10.3390/jcdd12070260 (PMC12295922; doi:10.3390/jcdd12070260)
Supplement: Supplementary file 1 [file jcdd-12-00260-s001.zip › jcdd-3590191-supplementary.pdf]

Supplementary Table S1. Quality Assessment of Included Studies Using the Newcastle–Ottawa Scale (NOS)

Study	Selection (0–4)	Comparability (0–2)	Outcome/Exposure (0–3)	Total Score (0–9)
Drca et al. (2023)	4	2	3	9
Molina et al. (2008)	4	2	3	9
Wilhelm et al. (2011)	3	1	2	6
Gabrielli et al. (2019)	3	1	2	6
Wilhelm et al. (2012a)	4	2	3	9
Wilhelm et al. (2012b)	4	2	3	9
Clauss et al. (2016)	3	1	2	6
Fernández et al. (2019)	3	1	2	6
Ujka et al. (2017)	3	1	2	6
Cipriani et al. (2020)	3	1	2	6
Ragab et al. (2023)	4	2	3	9
Picco et al. (2021)	3	1	2	6
Contreras-Briceño et al. (2021)	3	1	2	6
Jee et al. (2013)	3	1	2	6
Konwerski et al. (2021)	3	1	2	6
Li et al. (2023)	4	2	3	9
Breuckmann et al. (2009)	4	2	3	9
Shin et al. (2018)	3	1	2	6
Doni et al. (2022)	3	1	2	6
Haeusler et al. (2012)	4	2	3	9
Louis-Georges et al. (2019)	2	0	1	3
Madalosso & Raviele (2008a)	2	0	1	3
Madalosso & Raviele (2008b)	2	0	1	3
Maqueda et al. (2015)	2	0	1	3
Martínez-Mas et al. (1994)	2	1	1	4
Möckel et al. (1992)	3	1	2	6
Mont et al. (2009)	3	1	2	6
Störk et al. (1992)	3	1	2	6
Ujka et al. (2016)	3	1	2	6
